# Special Analytical and Health Commission on Tinned and Preserved Food

**Published:** 1906-09-08

**Authors:** 


### SPECIAL ANALYTICAL AND HEALTH COMMISSION ON TINNED AND PRESERVED FOOD.

### IX. CONDIMENTS—PRESERVED FISH—PRESERVED RABBIT.

In our last article we discussed fully the important part played by appetite in the digestion and assimilation of food. We also dilated upon the stimulating effect upon appetite of certain sauces and extracts. We must now pause to consider in detail the action and active principles of the more important appetisers.

### Salt and Saltness.

Common salt or sodium chloride holds a most unique position in the physiology of every-day life. One of the four fundamental tastes which we are capable of appreciating is that of saltness; and saltness in our spectrum of taste occupies as important and as central a position as green in our spectrum of colour. Without salt we cannot live. In so far as it promotes the secretion of the digestive juices by virtue of its taste, salt may be regarded as a condiment; but in that both its ions are essential mineral constituents of the fluids of the body, and also in so far as by virtue of its raising the osmotic pressure of our tissue fluids it may be a source of energy to the body, it occupies a peculiar position. The average person consumes approximately rather more than half an ounce of salt a day; in some cases this amount is greatly exceeded. The influence of salt upon health has been a matter of much observation and reasoning, but in spite of this no very clear facts with regard to it have been elicited. Salt is one of the most soluble of the mineral constituents of our food, and if the blood should happen at any time to contain as much solid material as it can keep in solution the ingestion of an extra quantity of salt would probably occasion the precipitation from it of some of its less soluble constituents. This seems certainly to be the case with some of the partially oxidised products of nitrogenous metabolism, *e.g.*, sodium biurate, a substance the deposition of which in the tissues is supposed to be associated with gout. Hence in the gouty diathesis we as a rule restrict the consumption of salt.

Ordinary salt on account of the faculty it has for attracting water is apt to become damp and difficult to manipulate, hence, if salt cannot be kept in a very dry place it is often advisable to mix with it some less hygroscopic substance. The one usually chosen is phosphate of soda which, though not so salt as the chloride, makes when mixed with the latter a salt powder which does not cake and does not attack silver.

### Other Mineral Condiments.

In addition to salt we possess other condiments belonging to the mineral kingdom, amongst which we may mention oxalic acid and its salts. This substance is present and gives flavour to or accentuates the flavour of certain articles of food, especially pepper, which contains .32 per cent.; sorrel, which contains .36 per cent.; and rhubarb, which contains .24 per cent.

### Physiological Action of Condiments.

In addition to the stimulating action of condiments upon the secretion of digestive juices they have two other important actions in digestion. Most of them have for their active principle a so-called volatile oil. Chemically the volatile oils belong to a class of substance known as the terpenes or a special class of hydrocarbons. These substances are all antiseptics. We may for the purposes of this article compare the stomach to a brewer's vat, *i.e.* to a vessel in which we wish a definite kind of fermentation to take place. The fermentation which we wish to take place in the stomach is that produced by the action of pepsin upon proteids—viz. proteolysis. For this fermentation the stomach is specially adapted both chemically and physiologically and in an ideal stomach this is the only kind of fermentation or digestion which should take place. Unfortunately there is continually swallowed with the food, or in some way introduced from without, other fermentative agents which attack those constituents of the gastric contents which they are capable of splitting up, and cause thereby the production of gas, the formation of local irritants and it may be generally poisonous, substances, and finally occasion a food waste to the body. This phenomenon may be described as secondary fermentation, and the ferments which produce it are unwelcome guests. In the natural gastric juice we have an agency which is capable, within certain limits, of destroying these stray ferments, but it often requires reinforcement. Powerful aid is given by condiments and sauces containing condiments.

The most perfectly functioning stomach is not capable of digesting all the contents of a mixed meal. If after such a meal we are to feel comfortable our stomach must not only digest what it can, but when it has done this it must pass on that food which it cannot digest into the duodenum, where the potentiality for further digestion exists. A very fertile source of indigestion is a sluggishly-moving stomach. Any substance which has the power of making the stomach contract or empty itself we term a carminative. Practically all the condiments we possess are carminatives, and hence in this second way facilitate digestion.

### Mustard, Pepper, and Vinegar.

The most frequently used condiments in this country are mustard, pepper, and vinegar. The active principle of mustard is a volatile oil. This volatile oil does not exist in mustard naturally, but when the latter substance is moistened with water a ferment (myrosin) splits up a glucoside (sinigrin) which mustard contains, and one of the products of this decomposition is allyl-isosulphocyanate or volatile oil of mustard. This reaction is a biological one and hence will only take place at biological temperatures, and this must be remembered by the cook and by the nurse, neither of whom must mix mustard with boiling water, or its yield in active principle will be very disappointing. Pepper, or rather black pepper, contains as its active condiment agency a basic substance known as piperine and a pungent resin. The active principle of vinegar is acetic acid. Ordinary vinegar should contain about 5 per cent. of acetic acid. Vinegar should be produced from the fermentation of alcoholic liquids by a definite variety of fungus—namely, mycoderma aceti. The alcoholic liquid thus fermented is derived from various sources. Good white vinegar should be obtained from the fermentation of white wine; good red vinegar from that of red wine. Such vinegars possess in addition to their acetic acid other substances derived from the wine, which give them a special bouquet and endow them no doubt with additional qualities as condiments. Another variety of vinegar, much used at the present day, is malt vinegar, which should be produced exclusively from the fermentation of malt liquors. Vinegar, by virtue of its dilute acidity, is a marked stimulant of salivary secretion, and greatly facilitates insalivation or the proper mastication of food. The complete reduction of food to a pulp in the mouth is a matter of great importance, not only in that such food is more easily digested than food swallowed in lumps, but it also appears from the most interesting work of Horace Fletcher that the amount of food necessary for a man doing a day's work can be greatly reduced if the ingested food be slowly masticated and thoroughly insalivated. We have seen in earlier articles of this series that the Voit figures for the amount of large calories necessary for a working man was 3,000. This can be reduced by prolonged insalivation, according to Horace Fletcher, to 1,500, and thus a tremendous economy in nutrition attained. In addition to its action as a condiment, vinegar, by virtue of its acetic acid, has a distinctly softening action upon meat fibre, and hence facilitates considerably the digestion of tough fibre, such, for instance, as that of crab and lobster, with which it is almost universally taken. In this article we have only been able to consider the condiments commonly used in this country and those which form the basis of most of our sauces. In the East, however, the number of condiments used in ordinary culinary processes is very large, and frequently includes substances which we regard as nauseating, and may even use in medicine as emetics, such, for instance as asafœtida and fennel.

### Pickles.

We have said nothing in our general remarks about pickles, but they essentially come under the consideration of condiments. They consist of small or chopped vegetables prepared in sauce, the basis of which is formed of the substances described above in varying proportions. The vegetables chosen are, as a rule, those which themselves contain volatile oils or substances of the nature of condiments and thus have a carminative action, such, for instance, as chillies, onions, small cucumbers, etc.

Although the above substances form the essential basis of digestive sauces and relishes, it must not be supposed for a moment that the making up of a sauce is an easy matter.

### Samples Received.

The various samples of sauces, relishes, and pickles which we have received we considered fully in a former article. Since, however, concluding our remarks upon the respective class of foods, several samples have been submitted to us and these we purpose considering at the present time.

### Tinned Fish.

Messrs. Richard Dickeson and Co., the well-known Army contractors, have kindly sent us a can of their preserved lobster. We have examined this preparation carefully, and have come to the conclusion that it is thoroughly sound and wholesome. The lobster is a tasty as well as a nutritious fish, and the demand for canned lobster is very large. So long as contractors supply articles as carefully prepared as the one before us, we think the public taste should be encouraged. We have also received from the same firm a sample of their canned salmon and their canned herrings. We have elsewhere pointed out that probably the greatest possible nutritional value for the expenditure of a given sum is to be obtained in preserved fish. Of all fish the herring is perhaps the cheapest, and the supply of it is spasmodic above all others, and hence its conservation is ethically most justifiable. The canned fresh herrings under consideration have an excellent taste and appearance, and a minute inspection of them shows that they are prepared in a thoroughly hygienic manner. The specimen of salmon is also good and wholseome. Messrs. Dickeson and Company have also submitted to us a sample of their canned boiled rabbit. The rabbit may be regarded as the chicken of the poor, and is probably, excluding fish, the cheapest variety of animal food in existence. We have no hesitation in recommending the preparation of rabbit under consideration.

